# Design, Synthesis and In Vitro Characterization of Novel Antimicrobial Agents Based on 6-Chloro-9*H*-carbazol Derivatives and 1,3,4-Oxadiazole Scaffolds

**DOI:** 10.3390/molecules25020266

**Published:** 2020-01-09

**Authors:** Alexandra T. Bordei Telehoiu, Diana C. Nuță, Miron T. Căproiu, Florea Dumitrascu, Irina Zarafu, Petre Ioniță, Carmellina D. Bădiceanu, Speranța Avram, Mariana C. Chifiriuc, Coralia Bleotu, Carmen Limban

**Affiliations:** 1 Department of Pharmaceutical Chemistry, Faculty of Pharmacy, “Carol Davila” University of Medicine and Pharmacy, 6 TraianVuia, 020956 Bucharest, Romania; alexandratelehoiu@yahoo.com (A.T.B.T.); diananuta@yahoo.com (D.C.N.); bcarmellina@yahoo.com (C.D.B.); carmen_limban@yahoo.com (C.L.); 2 Center for Organic Chemistry “C.D. Nenitzescu”, Romanian Academy, 202B Spl. Independentei, 060023 Bucharest, Romania; 3 Department of Organic Chemistry, Biochemistry and Catalysis, Faculty of Chemistry, University of Bucharest, 4-12 Regina Elisabeta, 030018 Bucharest, Romania; zarafuirina@yahoo.fr (I.Z.); p_ionita@yahoo.co.uk (P.I.); 4 Department of Anatomy, Animal Physiology and Biophysics, Faculty of Biology, University of Bucharest, 1-3 Ale. Portocalelor, 060101 Bucharest, Romania; speranta.avram@gmail.com; 5 Research Institute of the University of Bucharest (ICUB) and Microbiology Immunology Department, Faculty of Biology, University of Bucharest, 1-3 Ale. Portocalelor, 060101 Bucharest, Romania; carmen.chifiriuc@bio.unibuc.com; 6 Ștefan S. Nicolau Institute of Virology, Romanian Academy, 285 Mihai Bravu Avenue, 030304 Bucharest, Romania; cbleotu@yahoo.com

**Keywords:** oxadiazole, carbazole, carprofen, antimicrobial, cytotoxicity, in silico

## Abstract

In this paper, we aimed to exploit and combine in the same molecule the carbazole and the 1,3,4-oxadiazole pharmacophores, to obtain novel carprofen derivatives, by using two synthesis pathways. For the first route, the following steps have been followed: (i) (*RS*)-2-(6-chloro-9*H*-carbazol-2-yl)propanonic acid (carprofen) treatment with methanol, yielding methyl (*RS*)-2-(6-chloro-9*H*-carbazol-2-yl)propanoate; (ii) the resulted methylic ester was converted to (*RS*)-2-(6-chloro-9*H*-carbazol-2-yl)propane hydrazide (carprofen hydrazide) by treatment with hydrazine hydrate; (iii) reaction of the hydrazide derivative with acyl chlorides led to *N*-[(2*RS*)-2-(6-chloro-9*H*-carbazol-2-yl)propanoil]-*N*′-*R*-substituted-benzoylhydrazine formation, which; (iv) in reaction with phosphorus oxychloride gave the (*RS*)-1-(6-chloro-9*H*-carbazol-2-yl)-1-(1,3,4-oxadiazol-2-yl)ethane derivatives. In the second synthesis pathway, new 1,3,4-oxadiazole ring compounds were obtained starting from carprofen which was reacted with isoniazid, in the presence of phosphorus oxychloride to form (*RS*)-1-(6-chloro-9*H*-carbazol-2-yl)-1-[5-(4-pyridyl)-1,3,4-oxadiazol-2-yl]ethane. The synthesized compounds were characterized by IR, ^1^H-NMR and ^13^C-NMR, screened for their drug-like properties and evaluated for in vitro cytotoxicity and antimicrobial activity. The obtained compounds exhibited a good antimicrobial activity, some of the compounds being particularly active on *E. coli*, while others on *C. albicans*. The most significant result is represented by their exceptional anti-biofilm activity, particularly against the *P. aeruginosa* biofilm. The cytotoxicity assay revealed that at concentrations lower than 100 μg/mL, the tested compounds do not induce cytotoxicity and do not alter the mammalian cell cycle. The new synthesized compounds show good drug-like properties. The ADME-Tox profiles indicate a good oral absorption and average permeability through the blood brain barrier. However, further research is needed to reduce the predicted mutagenic potential and the hepatotoxicity.

## 1. Introduction

Carbazole and its derivatives represent an important group of aromatic heterocyclic compounds containing a nitrogen atom. Compounds containing this scaffold have important electronic and charge-transport properties, as well as a conjugated pi-electron system, which facilitate the easy introduction of various functional groups into the structurally rigid carbazole ring [1].

These properties result in the extensive applications of carbazole derivatives in the medical field, such as antitumor, antimicrobial, antihistaminic, antioxidant, anti-inflammatory, and psychotropic agents [2].

**Figure 1:**
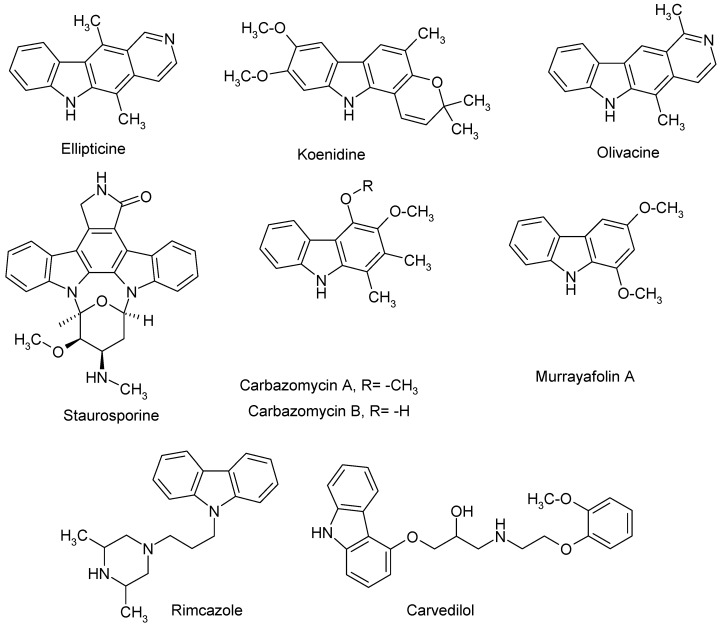
Carbazole derivatives with pharmacological properties.

The carbazole nucleus can be naturally found in the composition of some alkaloids as ellipticine [3], with antimalarial properties and koenidine, with potential antidiabetic effect [4], or staurosporine, with antibacterial, antifungal, and antitumoral activity [5], among them carbazomycins represent an unprecedented class of antibiotics with a carbazole heterocycle [6]. Carbazomycins A and B have proven to have antibacterial and antifungal activity, inhibiting the growth of phytopathogenic fungi, while Murrayafolin A, a compound isolated from *Murraya euchrestifolia Hayata* has shown a strong fungicidal effect on *Cladosporium cucumerinum* [7]. A new carbazole-derived alkaloid (1,8-dimethoxy-3-formylcarbazole) was isolated from the leaves of *Clausena heptaphylla*, being active against Gram-positive and Gram-negative bacteria, as well as fungi [8]. The carbazole acid ring is also present in many drugs, such as olivacine (antimalarial), rimcazole (antipsychotic and anticonvulsant), carvedilol (antihypertensive) [1,2] (Figure 1).

Some members of carbazole series have been shown to exhibit a broad spectrum of antifungal activity, acting as potent fungal plasma membrane proton adenosine triphosphatase inhibitors [9]. For example, 9-(9-ethyl-9*H*-carbazol-3-yl)-2-(phenoxy)acetamide derivatives, obtained by reacting 2-chloro-*N*-(9-ethyl-9*H*-carbazol-3-yl)acetamide with substituted phenols have shown antimicrobial activity [10]. Novel 5-[(9*H*-carbazol-9-yl)methyl]-*N*-[(substituted phenyl)(piperazin-1-yl)methyl]-1,3,4-oxadiazol-2-amines derivatives proved an antimicrobial effect and anti-proliferative activity, against the breast cancer cell line MCF7 [11].

Carprofen (2-(6-chloro-9*H*-carbazol-2-yl)propanoic acid) is a non-steroidal anti-inflammatory drug (NSAID), included in the class of propionic acid derivatives, that inhibits both COX-1 and COX-2 and is used commonly to combat inflammation and pain in animals with osteoarthritis or other inflammatory diseases. Carprofen was previously used in human medicine for over 10 years (1985–1995). It was generally well tolerated, with only mild side effects, such as gastro-intestinal pain and nausea. It is no longer marketed for human usage, after being withdrawn on commercial grounds, but is used by veterinarians as a supportive treatment for the relief of arthritic symptoms in geriatric dogs.

1,3,4-Oxadiazoles are heterocyclic compounds which have a diversity of useful biological effects including antibacterial [12], antifungal [13], analgesic [14], anti-inflammatory [15], antiviral [16], anticancer [17], antihypertensive [18], anticonvulsant [19], and anti-diabetic [20] properties.

**Figure 2:**
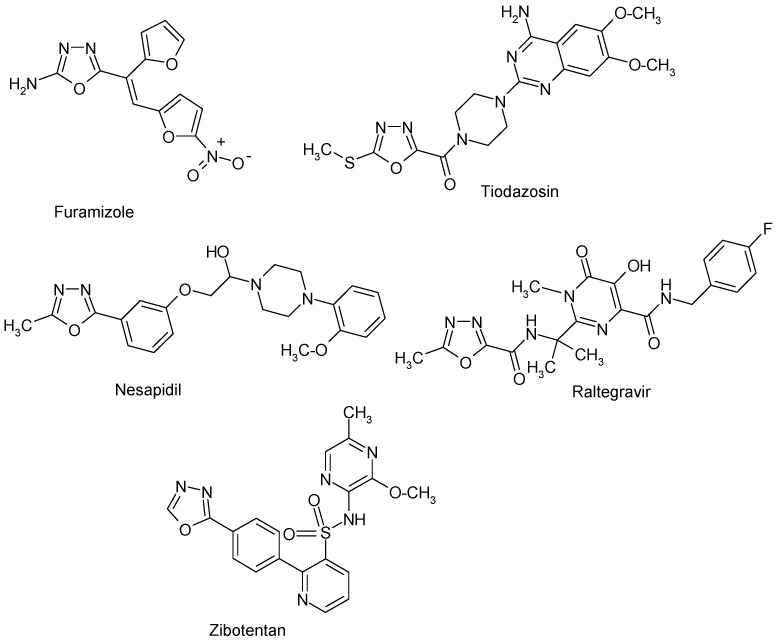
1,3,4-Oxadiazole derivatives used as drugs.

1,3,4-Oxadiazole nucleus is contained in the molecules of drugs such as: Furamizole (antibacterial), tiodazosin (an alpha-1 adrenergic antagonist), nesapidil (antihypertensive), raltegravir (antiretroviral), and zibotentan (anticancer) (Figure 2) [21].

The most commonly used synthetic route for the synthesis of 1,3,4-oxadiazoles includes the reactions of acid hydrazides with acid chlorides or carboxylic acids and cyclization of the formed acylhydrazines using a dehydrating agent, such as phosphorous pentaoxide, thionyl chloride, or phosphorous oxychloride [22].

In the present study, we aimed to combine in the same molecular framework the carbazole and the 1,3,4-oxadiazole pharmacophores, to obtain novel carprofen derivatives. The new synthesized compounds were characterized by IR, ^1^H-NMR and ^13^C-NMR, screened for their drug-like properties and evaluated for in vitro cytotoxicity and antimicrobial activity.

## 2. Results

### 2.1. Chemistry

**Scheme 1:**
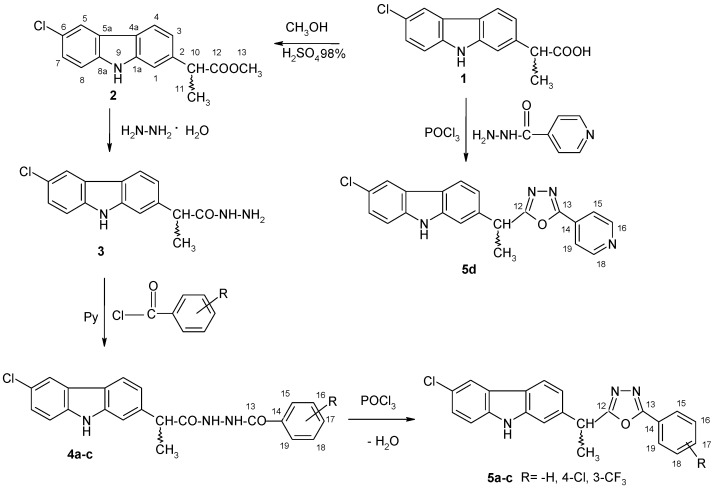
Synthesis pathways for the novel 2,5-disubstituted 1,3,4-oxadiazole **5a**–**d**.

The synthesis routes approached to obtain the target compounds are outlined in Scheme 1.

The reaction of (*RS*)-2-(6-chloro-9*H*-carbazol-2-yl)propanoic acid (carprofen) (**1**) with methanol, in the presence of concentrated sulfuric acid leading to methyl (2*RS*)-2-(6-chloro-9*H*-carbazol-2-yl)propanoate (carprofen methyl ester) (**2**). The reaction of compound **2** with hydrazine hydrate in refluxing ethanol gave (2*RS*)-2-(6-chloro-9*H*-carbazol-2-yl)propane hydrazide (carprofen hydrazide) (**3**).

The *N*,*N*′-disubstituted hydrazines (**4a**–**c**) were obtained by stirring the carprofen hydrazide (**3**) and the acyl chloride at room temperature in anhydrous pyridine.

The IR spectrum of compounds **4a**–**c** showed characteristic absorption band at 3201–3215 cm^−1^ due to N–H group and also to carbonyl absorption bands at 1706–1702 and 1649–1651 cm^−1^. Its ^1^H-NMR spectrum revealed singlets at δ 10.15–10.19 and 10.34–10.66 ppm due to two NH protons of the hydrazide moiety, in addition to singlet at δ 11.38–11.41 ppm assignable for -NH- proton of the carbazole core. The ^13^C-NMR spectral data for compounds **4a–c** revealed signals in the aromatic region and additional two singlets around δ 172.74–172.90 and in the region 164–165.93 ppm for the two carbonyl groups, which are in agreement with the assigned structures.

For the synthesis of 2,5-disubstituted 1,3,4-oxadiazoles (**5a**–**c**) the corresponding acylhydrazide was heated on the water bath with phosphorus oxychloride.

Starting from carprofen we also obtained (*RS*)-1-(6-chloro-9*H*-carbazol-2-yl)-1-[5-(4-pyridyl)-1,3,4-oxadiazol-2-yl]ethane (**5d**) by reaction with isoniazid, in the presence of phosphorus oxychloride.

The IR spectrum of compounds **5a**–**d** showed absorption bands in the region 3238–3268 cm^−1^ due to NH group. Its ^1^H-NMR spectrum displayed a doublet band at δ 1.80–1.90 ppm and quartet at 4.58–4.80 ppm corresponding to -CH_3_ and -CH- groups, respectively. The presence of the ethanyl group was also confirmed by signals around δ 19.50–20.04 and 36.98–38.01 ppm in the ^13^C-NMR spectrum of these compounds, which correspond to -CH_3_ and -CH- groups, respectively.

### 2.2. Biological Assays

The tested compounds presented MIC values ranging from 0.625 to 10 mg/mL (Table 1). The most susceptible microbial strains were the Gram-negative bacteria, followed by the fungal *C. albicans* strain. The best antibacterial effect was recorded for **4a** against *E. coli*, with MIC of 1.25 mg/mL and for **5c** against *C. albicans*, with MIC of 0.625 mg/mL.

The minimal bactericidal concentration (MBC) values ranged in the same interval as the MIC ones, being equal or one time higher than that corresponding MIC values, suggesting a microbicidal mechanism of action (Table 2).

All tested compounds exhibited excellent anti-biofilm properties against the bacterial and fungal strains, with Minimal Biofilm Eradication Concentration (MBEC) values generally ranging from 0.009 to 2.5 mg/mL (Table 3). The MBEC values were in some cases up to 256 times lower than MIC or MBC (Table 3). The highest susceptibility to all tested compounds was recorded for the *P. aeruginosa* biofilm.

**Figure 3:**
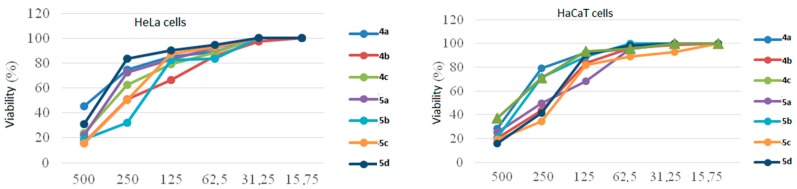
The cytotoxicity of compounds **4a**–**c**, **5a**–**d** on HeLa and HaCaT cells (evaluation at 48 h, using CellTiter 96^®^ AQ_ueous_ One Solution Cell Proliferation).

**Figure 4:**
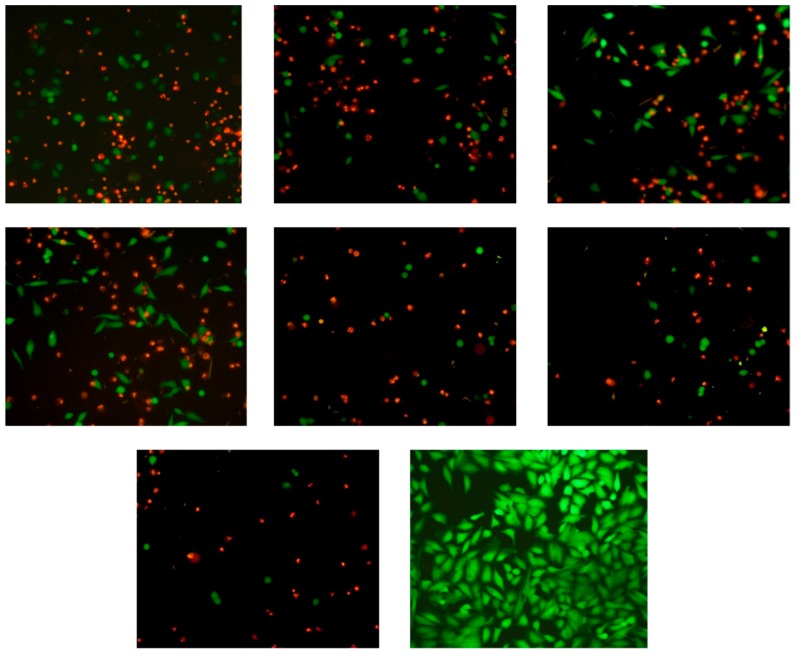
Viability of HeLa cells treated with 1mg/mL of **4a**–**c**, **5a**–**d** for 24 h. FDA/PI staining, 200×.

For the in vitro cytotoxicity assay, the results show that the tested compounds become toxic at concentrations higher than 100 μg/mL, with the HeLa cells and HaCaT cells viability gradually decreasing after 48 h of incubation, in a dose-dependent manner (Figure 3). All tested compounds are highly toxic at 1 mg/mL, the cellular viability decreasing dramatically after 24 h of incubation (Figure 4).

**Figure 5:**
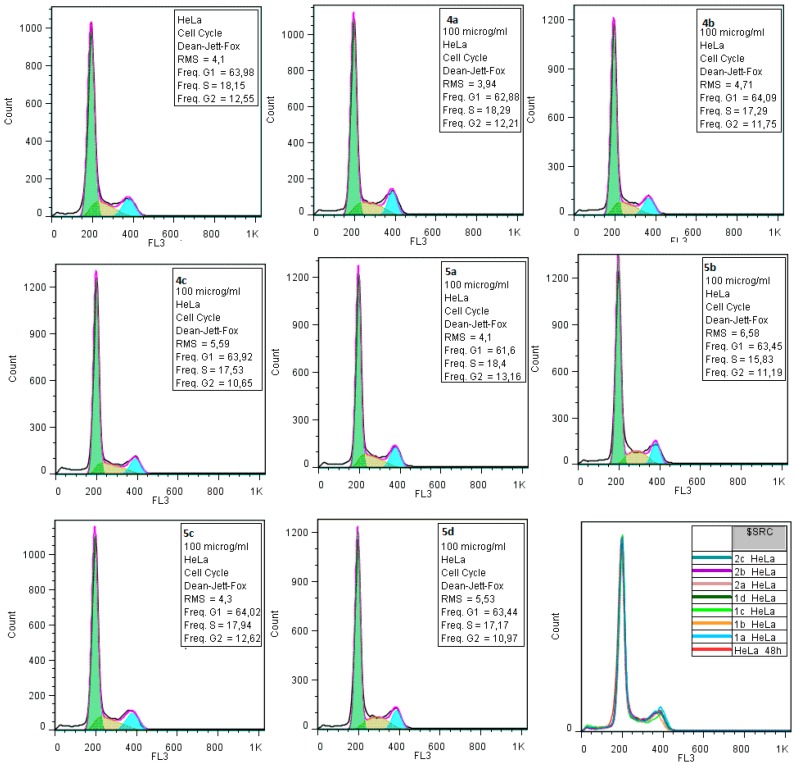
Flow cytometry histograms showing the influence of compounds **4a**–**c**, **5a**–**d** on the cellular cycle of HeLa and HaCaT cells. Y axis—intensity of fluorescence; X axis—the relative number of cells (15,000 events were investigated).

After 48 h, at 100 μg/mL, the compounds do not alter cell cycle phases of HeLa cells, but a fluctuation of the synthesis (S) phase is observed for HaCaT cells (Figure 5).

### 2.3. In Silico Analyses

The tested compounds comply with Lipinski rule and are predicted to have a good bioavailability according to the Veber rule (Table 4).

Regarding the predicted structural similarity of compounds **4a**–**c** and **5a**–**d** with other already known chemicals compounds, we noticed that compounds 1a, c recorded a higher similarity with compound 2-(6-chloro-9*H*-carbazol-2-yl)-*N*-phenylpropanamide, with biological activity, i.e., inhibition of Faah—fatty acid amide hydrolase active site (Table 5).

Considering that the structural similarities may induce similarities in biological activities, we identified the possible structural similarities of the analyzed compounds with other ligands (Table 5). Interesting results regarding structural similarity were observed for compounds 5a–c. While compounds **5a** and **5b** are similar with nociceptive receptor ligand 2-[2-[5-[2-(4-clorofenil)etil]-1,3,4-oxadiazol-2-il]-1~{*H*}-indol-4-il]-~{*N*},~{*N*}-dimetiletanamine, compound **5c** is similar with 2-(3~{*H*}-benzimidazol-5-yl)-5-[2-[4-(trifluoromethyl)phenyl]ethyl]-1,3,4-oxadiazole, a cyclotransferase inhibitor.

For compound **5d** we founded a poor similarity with other know compounds (Table 5).

Next, ADMET predictions suggest that all molecules have good intestinal absorption, with a value of more than 30% [23] (Table 6).

The Caco2 cell line permeability suggesting if a compound is absorbed after oral administration revealed a value greater than 0.9 for all of the tested molecules [23]. The **5a**, **5b**, and **5c** compounds have also shown an average blood brain barrier permeability. No drug–drug interactions affecting the renal clearance have been recorded for the tested compounds (Table 6).

The AMES tests that predicts the mutagenic potential [23] proved to be positive for all tested compounds (Table 7).

The maximum tolerated predicted dose for humans is considered to be low for all the tested molecules, as shown in Table 7. The inhibition of potassium channels encoded by the hERG gene is the main cause of the development of QT interval prolongation, on the electrocardiogram. Predictions in the pkCSM database suggest that all molecules inhibit hERG II, and those in the admetSAR database suggest that all molecules have poor inhibitory activity on hERG I (Table 7). In terms of hepatotoxicity, predictions suggest that the studied molecules exhibit hepatotoxicity.

## 3. Discussion

We synthesized novel derivatives based on 6-chloro-9*H*-carbazol and 1,3,4-oxadiazol scaffolds. The chemical structures of the novel compounds (carprofen hydrazide, *N*,*N*’-disubstituted hydrazine and 1,3,4-oxadiazoles derivatives) and the carprofen methyl ester were confirmed on the basis of spectral studies.

The biological activity data have shown that the synthesized compounds have a good antimicrobial activity, especially on Gram-negative strains, **4a** showing the best activity against *E. coli*. In contrast, **5c** is a potentially antifungal agent. It is to be highlighted that the obtained compounds acted preferentially on biofilm embedded cells, as revealed by the very low MBEC values obtained for some compound-microbial strain pairs, in comparison with the MIC and MBC values. Also, it is to be noticed the very good anti-biofilm activity of all tested compounds against the *P. aeruginosa* biofilm, well known for its high resistance to all currently available antimicrobial agents. The in vitro cytotoxicity of the tested compounds performed on two cellular lines (i.e., HeLa and HaCaT cells) reveal a dose- and time-related effect, the cellular viability decreasing with the increase of concentration (>100 μg/mL) and incubation time (48 h). However, at concentrations lower than 100 μg/mL, the tested compounds do not induce cytotoxicity and do not alter the mammalian cell cycle.

The new synthesized compounds show good drug-like properties. All studied compounds comply with Lipinski rule and also, have a good predicted bioavailability according to the Veber rule.

To predict a possible biological activity of compounds **4a**–**c** and **5a**–**d**, we evaluated by bioinformatics tools the molecular similarity of our compounds and other compounds, already included into most popular databases. Our results have shown that compounds **4a**–**c** exhibit a molecular similarity with Faah inhibitors, while the compounds **5a**–**c**, appeared to have nociceptive and QPCT—glutaminyl-peptide cyclotransferase inhibitory activities.

The organic cation transporter OCT2 is involved in the renal excretion of cationic drugs and raises the possibility of drug–drug interactions between an inhibitor and a substrate drug, which will have a decreased renal clearance [24].

The ADMET predictions show that the compounds may have good oral absorption. Plus, **5a**, **5b**, and **5c** show an average permeability through the blood brain barrier. Unfortunately, the compounds may also have mutagenic potential and increased hepatotoxicity.

Research may continue in this direction, by synthesizing similar compounds with lower cytotoxic, mutagenic and hepatotoxic potential.

## 4. Materials and Methods

### 4.1. Measurements

Melting points were determined in open glass capillary tube on an Electrothermal 9100 (Bibby Scientific Ltd., Stone, UK) capillary melting point apparatus and are uncorrected.

The FT-IR ATR (attenuated total reflection Fourier transform infrared) spectra were taken in solid state on a Bruker Vertex 70 spectrophotometer (Bruker Corporation, Billerica, MA, USA).

The ^1^H-NMR and ^13^C-NMR spectra were recorded in dimethylsulfoxide-d6 (DMSO-*d*_6_) or deuterated chloroform on a Bruker Fourier 300 MHz or Bruker AvanceIII 500 MHz instrument (Bruker Corporation, Billerica, MA, USA), using tetramethylsilane as the internal standard, the chemical shifts are expressed in δ ppm.

The ^1^H-NMR data are reported in the following order: chemical shifts, multiplicity, the coupling constants, number of protons and signal/atom attribution. The apparent resonance multiplicity is described as s (singlet), d (doublet), q (quartet), m (multiplet), dd (double doublet), and br (broad) signal.

For the ^13^C-NMR data the order is the following: chemical shifts and signal/atom attribution.

The mass spectrum of compound **5d** was recorded on a Maxis Bruker 4G spectrometer (Bruker Corporation, Billerica, MA, USA) with an electrospray ionization source (ESI). The samples were dissolved in DMSO 1 mg/1 mL and then the solution was diluted with methanol to a final concentration of 1 μg/1 mL. The scanning range of molecular ions (*m*/*z*) was 50–1250.

### 4.2. Chemical Synthesis and Spectral Characterization

*Methyl (2RS)-2-(6-chloro-9H-carbazol-2-yl)propanoat (carprofen methyl ester)* (**2**)

(*RS*)-2-(6-Chloro-9*H*-carbazol-2-yl)propanoic acid (carprofen) (10 g, 0.037 mol) was dissolved with stirring in 400 mL methanol absolute. Then 1.2 mL concentrated sulfuric acid (98%) was dropwise added as a catalyst and stirring was continued at room temperature for 8 h. The reaction mixture was left overnight at room temperature, and then the methanol was evaporated at low pressure until a precipitate appeared. Over the reaction mixture, 200 mL of water was added and the precipitate was filtered off at low pressure and washed well with water on the filter to remove the acid traces. The white or slightly yellow precipitate was air dried for 24 h.

We obtained 10 g (yield 95%) of carprofen methyl ester (m.p. 107–110 °C).

FT-IR (solid in ATR, ν cm^−1^): 3450w; 3405vs; 2983w; 2944w; 2875w; 1729vs; 1697m; 1624m; 1448m; 1429m; 1325w; 1269w; 1236w; 1193m; 1172m; 1093w; 1059m; 873w; 806m; 731w; 695w.

^1^H-NMR (CDCl_3_, δ ppm, *J* Hz): 8.26 (brs, 1H, H-9); 7.96 (brs, 1H, H-5); 7.92 (d, 1H, *J* = 8.1 Hz, H-4); 7.33 (d, *J* = 8.6 Hz, 1H, H-8); 7.31 (brs, 1H, H-1); 7.25 (d, *J* = 8.1 Hz, 1H, H-3); 7.17 (d, 1H, H-7, 8.6); 3.90 (q, *J* = 7.1 Hz, 1H, H-10); 3.71 (s, 3H, H-13); 1.60 (d, *J* = 7.1 Hz, 3H, H-11);

^13^C-NMR (CDCl_3_, δ ppm): 175.49 (C-12); 140.29 (C-8a); 139.01 (C-1a); 138.00 (C-2); 125.70 (C-7); 124.74 (C-5a); 124.11 (C-4a); 121.53 (C-6); 120.54 (C-4); 119.84 (C-5); 119.47 (C-3); 111.54 (C-8); 109.49 (C-1); 52.17 (C-13); 45.74 (C-10); 18.86 (C-11).

*(2RS)-2-(6-chloro-9H-carbazol-2-yl)propanohydrazide (carprofen hydrazide)* (**3**)

Carprofen methyl ester (6 g, 0.021 mol) in ethanol 96% or absolute (40 mL) is treated under magnetic stirring, with hydrazine hydrate 100% (7 mL) and refluxed for 8 h with continuous stirring. Then the mixture is cooled and the carprofen hydrazide is filtered off at low pressure and washed with cold alcohol on the filter; 4.2 g of white carprofen hydrazide are obtained with a melting point of 241–243 °C (yield 70%).

FT-IR (solid in ATR, ν cm^−1^): 3347s; 3260m; 2979w;2873w; 1632vs; 1517m; 1462s; 1428m; 1379w; 1338m; 1269m; 1238s; 1120w; 1061m; 988m; 926w; 885m; 828w; 800m; 733w; 690w.

^1^H-NMR (300 MHz, dmso-*d*_6_, δ ppm, *J* Hz): 11.36 (s, 1H, H-9); 9.24 (s, 1H, HN); 8.15 (d, *J* = 2.2 Hz, 1H, H-5); 8.05 (d, *J* = 8.2 Hz, 1H, H-4); 7.48 (d, *J* = 8.5 Hz, 1H, H-8); 7.47 (brs, 1H, H-1); 7.35 (dd, *J* = 2.2 Hz, *J* = 8.5 Hz, 1H, H-7); 7.16 (dd, *J* = 1.4 Hz, *J* = 8.2 Hz, 1H, H-3); 4.21 (brs, 2H, H-N); 3.70 (q, *J* = 6.9 Hz, 1H, H-10); 1.42 (d, *J* = 6.9 Hz, 3H, H-11);

^13^C-NMR (75 MHz, dmso-*d*_6_, δ ppm): 173.05 (C-12); 140.67 (C-8a); 140.56 (C-1a); 138.36 (C-2); 123.71 (C-5a); 123.56 (C-4a); 122.85 (C-6); 125.02 (C-7); 120.34 (C-4); 119.64 (C-5); 118.90 (C-3); 112.34 (C-8); 109.71 (C-1); 43.81 (C-10); 18.79 (C-11).

*N-[(2RS)-2-(6-chloro-9H-carbazol-2-yl)propanoyl]-N’-benzoylhydrazine* (**4a**)

In a round bottomed flask equipped with stirrer, carprofen hydrazide (0.86 g, 0.003 mol) and benzoyl chloride (0.42 g, 0.003 mol) and anhydrous pyridine (20 mL) are introduced. The mixture was stirred at room temperature for 4 h. After the reaction time has expired, the mixture was poured into a dilute, cold solution of hydrochloric acid (40 mL 10% HCl solution). A precipitate was obtained, isolated by filtration, washed on the filter with water and used in the steps in the raw state.

After drying, 1.02 g of *N*,*N*’-disubstituted hydrazine were obtained, with a melting point (m.p.) of 262–263 °C at a yield of 87% toward hydrazide. The compound is soluble at room temperature in pyridine, dimethylsulfoxide, dimethylformamide, after heating in methanol, ethanol, isobutanol, and hardly soluble after heating in isopropanol, xylene, insoluble in ethyl acetate, hexane and water.

FT-IR (solid in ATR, ν cm^−1^): 3356s; 3215m; 3084w; 3024w; 2974w; 2934w; 1706vs; 1651vs; 1576m; 1466s; 1367w; 1273m; 1241m; 1180w; 1144w; 1066w; 947w; 890w; 804w; 739w; 705m; 647w; 601m.

^1^H-NMR (300 MHz, dmso-*d*_6_, δ ppm, *J* Hz): 11.38 (brs, 1H, H-9); 10.34 (s, 1H, HN); 10.15 (s, 1H, NH); 8.17 (d, *J* = 1.5 Hz, 1H, H-5); 8.09 (dd, *J* = 1.5 Hz, *J* = 8.1 Hz, 1H, H-7); 7.86 (d, *J* = 8,1 Hz, 2H, H-15, H-19); 7.58- 7.44 (m, 4H, H-4, H-16, H-17, H-18); 7.53 (sl, 1H, H-1); 7.35 (d, *J* = 8.1 Hz, 1H, H-8); 7.23 (d, *J* = 8.4 Hz, 1H, H-3); 3.92 (q, *J* = 6.9 Hz, 1H, H-10); 1.50 (d, *J* = 6.9 Hz, 3H, H-11);

^13^C-NMR (75 MHz, dmso-d6, δ ppm): 172.74 (C-12); 165.93 (C-13); 140.28 (C-8a); 138.91 (C-1a); 138.11 (C-2); 132.32 (C-14); 131.70 (C-17); 128.35 (C-16, C-18); 127.30 (C-15, C-19); 124.93 (C-7); 123.53 (C-5a); 122.72 (C-4a); 120.30 (C-4); 120.26 (C-6); 119.56 (C-5); 118.89 (C-3); 112.20 (C-8); 109.72 (C-1); 43.56 (C-10); 18.85 (C-11).

*N-[(2RS)-2-(6-chloro-9H-carbazol-2-yl)propanoyl]-N’-(4-chlorobenzoyl)hydrazine* (**4b**)

The compound was prepared by the method described for (**4a**), from 4-chlorobenzoyl chloride (0.525 g, 0.003 mol).

There were obtained 1.04 g of compound (81% yield toward hydrazide), with m.p. 268–270 °C.

The compound is soluble at room temperature in pyridine, dimethylsulfoxide, dimethylformamide, after heating in isobutanol, hardly soluble after heating in methanol, ethanol, isopropanol, ethyl acetate, and insoluble in xylene, hexane and water.

FT-IR (solid in ATR, ν cm^−1^): 3414w; 3201m; 1599vs; 1567m; 1454m; 1273w; 1221w; 1152w; 1093w; 848w; 802w; 754w; 730w; 659w.

^1^H-NMR (500 MHz, dmso-*d*_6_, δ ppm, *J* Hz): 11.41 (brs, 1H, H-9); 10.46 (s, 1H, HN); 10.21 (s, 1H, NH); 8.17 (d, *J* = 2.1 Hz, 1H, H-5); 8.09 (d, *J* = 8.1 Hz, 1H, H-4); 7.87 (d, *J* = 8.1 Hz, 2H, H-15, H-19); 7.56 (d, *J* = 8,1 Hz, 2H, H-16, H-18); 7.52 (d, *J* = 1.3 Hz, 1H, H-1); 7.48 (d, *J* = 8.7 Hz, 1H, H-8); 7.36 (dd, *J* = 2.1 Hz, *J* = 8.7 Hz, 1H, H-7); 7.22 (dd, *J* = 1.3 Hz, *J* = 8.1 Hz, 1H, H-3); 3.93 (q, *J* = 6.9 Hz, 1H, H-10); 1.49 (d, *J* = 6.9 Hz, 3H, H-11);

^13^C-NMR (125 MHz, dmso-*d*_6_, δ ppm): 172.90 (C-12); 164.49 (C-13); 140.55 (C-8a); 139.98 (C-1a); 138.38 (C-2); 136.69 (C-17); 131.16 (C-14); 129.35 (C-15, C-19); 128.63 (C-16, C-18); 125.06 (C-7); 123.62 (C-4a); 122.83 (C-5a); 120.43 (C-4); 120.37 (C-6); 119.67 (C-5); 118.97 (C-3); 112.31 (C-8); 109.81 (C-1); 43.57 (C-10); 18.83 (C-11).

*N-[(2RS)-2-(6-chloro-9H-carbazol-2-yl)propanoyl]-N’-(3-trifluoromethylbenzoyl)hydrazine* (**4c**)

Following the synthesis procedure described for (**4a**), using of 3-trifluoromethylbenzoyl chloride (0.625 g, 0.003 mol), there were obtained 1.06 g of the compound, resulted after recrystallization from isopropanol:water 1:3, yield 77% toward hydrazide, m.p. 216–219 °C.

The compound is soluble at room temperature in pyridine, dimethylsulfoxide, dimethylformamide, ethyl acetate, methanol, ethanol, after heating in isopropanol, isobutanol, xylene and insoluble in hexane and water.

FT-IR (solid in ATR, ν cm^−1^): 3405m; 3357vs; 3209m; 3079w; 3023w; 2967w; 2932w; 1708vs; 1649vs; 1550w; 1474m; 1449m; 1331s; 1273m; 1225m; 1174s; 1125s; 1071m; 923w; 880w; 818w; 742w; 693m; 603m;

^1^H-NMR (500 MHz, dmso-*d*_6_, δ ppm, *J* Hz): 11.38 (s, 1H, HN-9); 10.66 (brs, 1H, HN); 10.29 (s, 1H, NH); 8.20 (brs, 1H, H-15); 8.17 (d, *J* = 1.9 Hz, 1H, H-5); 8.16 (d, *J* =8.1 Hz, 1H, H-19); 8.09 (d, *J* = 8.1 Hz, 1H, H-4); 7.95 (dl, *J* = 8.1 Hz, 1H, H-17); 7.75 (tl, *J* = 8.1 Hz, 1H, H-18); 7.53 (brs, 1H, H-1); 7.48 (d, *J* = 8.6 Hz, 1H, H-8); 7.36 (dd, *J* = 1.9 Hz, *J* = 8.6 Hz, 1H, H-7); 7.22 (dd, *J* = 1.3 Hz, *J* = 8.1 Hz, 1H, H-3); 3.94 (q, *J* = 7.0 Hz, 1H, H-10); 1.49 (d, *J* = 7.0 Hz, 3H, H-11);

^13^C-NMR (125 MHz, dmso-*d*_6_, δ ppm): 172.82 (C-12); 164.00 (C-13); 140.53 (C-8a); 139.94 (C-1a); 138.36 (C-2); 133.29 (C-14); 131.53 (C-19); 130.06 (C-18); 129.52 (q, *J*(3F-C^16^)=31.7 Hz, C-16); 128.43 (q, *J*(F-C^17^)=3.8 Hz, C-17); 125.02 (C-7); 124.05 (q, *J*(F-C^15^)=3.8 Hz, C-15); 123.88 (C-4a); 123.70 (q, *J*(3F-C)=267.8 Hz, -CF_3_); 123.63 (C-5a); 120.40 (C-6); 120.45 (C-4); 119.65 (C-5); 118.91 (C-3); 112.33 (C-8); 109.83 (C-1); 43.56 (C-10); 18.78 (C-11).

*(RS)-1-(6-chloro-9H-carbazol-2-yl)-1-(5-phenyl-1,3,4-oxadiazol-2-yl)ethane* (**5a**)

An amount of 0.784 g (0.002 mol) of corresponding acylhydrazide (**4a**) and 8 mL of phosphorus oxychloride were introduced in a round bottom flask equipped with a reflux condenser to which a calcium chloride tube was attached. The mixture was heated on the water bath for 9 h, then cooled and poured, under stirring, over ice water. The resulting precipitate was filtered off, washed with water and recrystallized from isopropanol:water 1:2.5.

An amount of 0.48 g of the compound is obtained, yield 64% toward acyl hydrazide with m.p. 185–186.5 °C, soluble at room temperature in pyridine, ethyl acetate, dimethylsulfoxide, dimethylformamide, after heating in methanol, ethanol, isopropanol, isobutanol, xylene and insoluble in hexane and water.

FT-IR (solid in ATR, ν cm^−1^):3238s; 2979w; 2940w; 2780w; 1609m; 1551s; 1454vs; 1375w; 1330w; 1268s; 1244m; 1210m; 1062m; 1017w; 925w; 887w; 816m; 775w; 752m; 685s; 649w; 621m.

^1^H-NMR (300 MHz, CDCl_3_, δ ppm, *J* Hz): 8.70 (brs, 1H, H-9, deuterable); 8.01- 7.94 (m, 4H, H-4, H-7, H-15, H-19); 7.52 - 7.39 (m, 4H, H-3, H-16, H-17, H-18); 7.33 (brs, 2H, H-1, H-5); 7.26 (d, *J* = 8.1 Hz, 1H, H-8); 4.58 (q, *J* = 7.2 Hz, 1H, H-10); 1.90 (d, *J* = 7.2 Hz, 3H, H-11);

^13^C-NMR (75 MHz, CDCl_3_, δ ppm): 169.17 (C-12); 165.10 (C-13); 140.51 (C-8a); 138.77 (C-2); 138.24 (C-1a); 131.71 (C-17); 129.00 (C-16, C-18); 126.87 (C-15, C-19); 125.96 (C-7); 124.90 (C-14); 124.10 (C-5a); 123.82 (C-4a); 121.91 (C-6); 120.89 (C-4); 119.98 (C-5); 119.18 (C-3); 111.75 (C-8); 109.58 (C-1); 38.01 (C-10); 20.04 (C-11).

*(RS)-1-(6-chloro-9H-carbazol-2-yl)-1-(5-(4-chlorophenyl)-1,3,4-oxadiazol-2-yl)ethane* (**5b**)

The synthesis followed the procedure described for the preparation of compound **5a**, using corresponding acyl hydrazide (**4b**) (0.819 g, 0.002 mol). Purification was done from isopropanol.

An amount of 0.56 g of the compound was obtained, yield 69% toward acyl hydrazide with m.p. 200–204 °C, soluble at room temperature in pyridine, ethyl acetate, dimethylsulfoxide, dimethylformamide, after heating in ethanol, isopropanol, isobutanol, xylene, hardly soluble after heating in methanol and insoluble in hexane and water.

FT-IR (solid in ATR, ν cm^−1^): 3268m; 3088w; 2978w; 1606s; 1563m; 1481vs; 1462s; 1432m; 1408w; 1338w; 1274s; 1246m; 1095m; 1063m; 1013m; 873w; 834m; 817s; 792m; 627w;

^1^H-NMR (300 MHz, dmso-*d*_6_, δ ppm, *J* Hz): 11.41 (s, 1H, H-9); 8.18 (d, *J* = 2.2 Hz, 1H, H-5); 8.13 (d, *J* = 8.2 Hz, 1H, H-4); 7.93 (d, *J* = 8.8 Hz, 2H, H-15, H-19); 7.61 (d, *J* = 8.8 Hz, 2H, H-16, H-18); 7.48 (d, *J* = 8.8 Hz, 1H, H-8); 7.46 (d, *J* = 1.3 Hz, 1H, H-1); 7.36 (dd, *J* = 2.2 Hz, *J* = 8.8 Hz, 1H, H-7); 7.18 (dd, *J* = 1.6 Hz, *J* = 8.2 Hz, 1H, H-3); 4.73 (q, *J* = 7.2 Hz, 1H, H-10); 1.79 (d, *J* = 7.2 Hz, 3H, H-11);

^13^C-NMR (75 MHz, dmso-*d*_6_, δ ppm): 169.18 (C-12); 163.39 (C-13); 140.57 (C-8a); 138.98 (C-1a); 138.42 (C-2); 136.61 (C-14); 129.56 (C-15, C-19); 128.22 (C-16, C-18); 125.33 (C-7); 123.46 (C-4a); 122.99 (C-5a); 122.21 (C-17); 121.14 (C-4); 120.82 (C-6); 119.77 (C-5); 118.38 (C-3); 112.44 (C-8); 109.83 (C-1); 36.95 (C-10); 19.91 (C-11).

*(RS)-1-(6-chloro-9H-carbazol-2-yl)-1-(5-(3-trifluoromethylphenyl)-1,3,4-oxadiazol-2-yl)ethane* (**5c**)

Following the synthesis procedure described for the preparation of compound **5a**, of the corresponding acyl hydrazide (**4c**) (0.919 g 0.002 mol) were used. Purification was done from isopropanol.

An amount of 0.55 g of compound was obtained, 62% toward acyl hydrazide, m.p. 169–172 °C, soluble at room temperature in pyridine, ethyl acetate, dimethylsulfoxide, dimethylformamide, after heating in methanol, ethanol, isopropanol, isobutanol, xylene and insoluble in hexane and water.

FT-IR (solid in ATR, ν cm^−1^): 3416w; 3239w; 3083w; 2984w; 1616w; 1559m; 1469m; 1428m; 1378w; 1328s; 1272m; 1244m; 1170m; 1131vs; 1068m; 1014w; 971w; 927w; 869w; 811m; 752w; 732w; 697m.

^1^H-NMR (500 MHz, dmso-*d*_6_, δ ppm, *J* Hz): 11.40 (brs, 1H, H-9); 8.22 (d, *J* = 7.9 Hz, 1H, H-19); 8.18 (brs, 1H, H-15); 8.17 (s, 1H, H-5); 8.13 (d, *J* = 8.1 Hz, 1H, H-8); 7.96 (brd, *J* = 7.9 Hz, 1H, H-17); 7.79 (t, *J* = 7.9 Hz, 1H, H-18); 7.48 (s, 1H, H-1); 7.48 (d, *J* = 8.5 Hz, 1H, H-4); 7.36 (dd, *J* = 2.1 Hz, *J* = 8.5 Hz, 1H, H-7); 7.19 (dd, *J* = 1.3 Hz, *J* = 8.1 Hz, 1H, H-3); 4.76 (q, *J* = 7.2 Hz, 1H, H-10); 1.80 (d, *J* = 7.2 Hz, 3H, H-11);

^13^C-NMR (125 MHz, dmso-*d*_6_, δ ppm): 169.54 (C-12); 163.08 (C-13); 140.43 (C-8a); 138.94 (C-1a); 138.29 (C-2); 130.85 (C-18); 130.44 (C-19); 130.08 (q, *J*(3F-C^16^)=32.5 Hz, C-16); 128.37 (q, *J*(3F-C^17^)=3.8 Hz, C-17); 125.35 (C-7); 124.45 (C-14); 123.60 (q, *J*(3F-C)=212.5 Hz, -CF_3_); 123.47 (C-4a); 123.43 (C-5a); 122.73 (q, *J*(3F-C^15^)=3.8 Hz, C-15); 121.14 (C-4); 120.84 (C-6); 119.78 (C-5); 118.42 (C-3); 112.41 (C-8); 109.82 (C-1); 36.98(C-10); 19.99 (C-11).

*(RS)-1-(6-chloro-9H-carbazol-2-yl)-1-[5-(4-pyridil)-1,3,4-oxadiazol-2-yl]ethane* (**5d**)

To a round bottom flask equipped with a stirrer bar and a condenser was introduced in sequence, POCl_3_ (2 mL), carprofen (0.273 g, 0.001 mol), and isoniazid (0.001 mol). The mixture was stirred for a few minutes and then heated under reflux for 2.5 h. Benzene or toluene (10 mL) was added and the mixture evaporated under reduced pressure on a rotary evaporator. The residue was treated with ice-water (10 g) and the red brick precipitate was filtered off under vacuum and washed several times with cold water (ice water). The orange product was crystallized from a mixture water/ethanol (2:1). 

An amount of 0.34 g compound (C_21_H_15_ON_4_Cl) was obtained, with yield 91% toward carprofen, m.p. 173–175 °C, soluble at room temperature in acetone, dimethylsulfoxide, dimethylformamide, and 1:1 dichloromethane: methanol, by heating in methanol, benzene, hexane, dichloromethane, dimethylsulphoxide, hardly soluble by heating in ethanol, chloroform, toluene, petroleum ether, and insoluble in xylene, ethyl ether, ethyl acetate and water.

FT-IR (solid in ATR, ν cm^−1^): 3241m; 3104w; 2979w; 2934w; 1691m; 1610w; 1551m; 1473vs; 1461vs; 1432m; 1358w; 1337w; 1273vs; 1217m; 1173m; 1065s; 928w; 871w; 817m; 753w; 699w; 584w.

^1^H-NMR (500 MHz, CDCl_3_, δ ppm, *J* Hz): 11.41 (s, 1H, H-9); 8.80 (dd, *J* = 1.6 Hz, *J* = 8.2 Hz, 2H, H-16, H-18); 8.19 (s, 1H, H-5); 8.13- 8.19 (m, 2H, H-15, H-19); 7.90 (d, *J* = 8.4 Hz, 1H, H-8); 7.49 (s, 1H, H-1); 7.48 (d, *J* = 8.5 Hz, 1H, H-4); 7.36 (dd, *J* = 1.8 Hz, *J* = 8.4 Hz, 1H, H-7); 7.18 (d, *J* = 8.5 Hz, 1H, H-3); 4.80 (q, *J* = 7.2 Hz, 1H, H-10); 1.80 (d, *J* = 7.2 Hz, 3H, H-11);

^13^C-NMR (125 MHz, CDCl_3_, δ ppm): 170.03 (C-12); 162.65 (C-13); 150.74 (C-16, C-18); 140.56 (C-8a); 138.79 (C-1a); 130.65 (C-2); 125.86 (C-7); 125.36 (C-14); 123.45 (C-4a); 123.0 (C-5a); 122.99 (C-4); 120.86 (C-6); 120.28 (C-15, C-19), 120.20 (C-5); 118.41 (C-3); 112.47 (C-8); 109.90 (C-1); 36.98 (C-10); 19.50 (C-11).

In order to confirm the structure, the compound **5d** was also analyzed by mass spectroscopy, revealing the appearance of a peak corresponding to the molecular ion 375.1 [M + H], which confirms the molecular weight of this compound.

### 4.3. Antimicrobial Activity Assay

The antimicrobial activity of the synthesized compounds was determined using three methods, against a panel of Gram-negative (*Escherichia coli* ATCC 25922, *Pseudomonas aeruginosa* ATCC 27853) and Gram-positive (*Staphylococcus aureus* ATCC 25923, *Enterococcus faecalis* ATCC 29212) bacteria, as well as the fungal strain *Candida albicans* ATCC 90029.

*Minimal inhibitory concentration (MIC) assay*: The minimum amount of the tested compounds that inhibited the microbial growth was evaluated using the microdilution method in liquid Mueller Hinton medium. The tested binary concentrations in the range of 5–0.009 mg/mL were achieved starting from a stock solution prepared in DMSO of 10 mg/mL, in a 100 µL culture medium final volume, seeded with a 20 µL microbial suspension of 0.5 MacFarland density. In each test, a microbial culture positive control and a sterile medium negative control were performed. The plates were incubated for 24 h at 37 °C. The results are shown in Table 1 [25,26,27].

*Minimal bactericidal concentration (MBC) assay*: after reading the MIC value, a volume of 10 µL from the well corresponding to the MIC value and from all the previous wells was seeded on solid PCA (plate count agar) medium to determine the MBC value, corresponding to the concentration at which the total inhibition of microbial growth on the solid medium was obtained (Table 2).

*Anti-biofilm activity assay*: the plates used to determine the MIC and MBC were washed two to three times with sterile saline to remove free and poorly adherent cells, after which the biofilms formed on the walls of the wells were fixed for 5 min with 100 μL of cold methanol, stained with 1% alkaline crystal violet solution for 15 min and then resuspended in acetic acid solution 33%. After homogenizing the colored suspension, its optical density was measured at 490 nm. The minimal biofilm eradication concentration (CMEB) was determined to be the lowest concentration of the tested compounds at which the decrease in absorbance value, measured at 490 nm, was observed in comparison to the positive control (Table 3) [28].

### 4.4. In Vitro Cytotoxicity Assay

The cell cultures used in this study were HeLa (ATCC^®^ CCL-2™) and the HaCaT immortalized keratinocyte line. They were maintained in DMEM: F12 medium supplemented with 10% fetal bovine serum.

The compounds **4a**–**c**, **5a**–**d** were diluted in DMSO at a concentration of 20 mg/mL and brought to a concentration of 2 mg/mL in DMEM: F12. The solutions were sterilized by using a 0.22 μm filter.

#### 4.4.1. Evaluation of Cytotoxicity Using CellTiter 96^®^ AQueous One Solution Cell Proliferation Assay

The cells were seeded at a density of 7.5 × 10^4^/well, in 96-well plates, in the presence of different concentrations of the tested compounds, ranging from 500 μg/mL to 15.75 μg/mL. After 48 h, the effect of the compounds was evaluated by the addition of tetrazolium salt, 3-(4,5-dimethylthiazol-2-yl)-5-(3-carboxymethoxyphenyl)-2-(4-sulfophenyl)-2*H*-tetrazolium. Toxicity assessment was performed at 2 h, by reading the absorbance at 490 nm, using the Tristar Berthold Technologies spectrophotometer (Berthold Technologies, Bad Wildbad, Germany).

#### 4.4.2. Toxicity Assessment Using Fluorescein Diacetate Staining (FDA)-Propidium Iodide (PI)

The cells were seeded at a density of 7.5 × 10^5^/well in 24-well plates. The medium contained 1 mg/mL compound. After 24 h, cell viability was assessed by staining with FDA (10 μg/mL) and PI (20 μg/mL). Cells were observed on the Zeiss fluorescence microscope (Gottingen, Germany).

#### 4.4.3. Evaluation of the Cellular Cycle

The cells were seeded at a density of 7.5 × 10^5^/well in 24-well plates and maintained in culture medium containing the tested compounds, at a concentration of 100 μg/mL, for 48 h. After incubation, the cells were trypsinized, washed in phosphate buffer saline, fixed in 70% cold ethanol and stained with 50 μg/mL propidium iodide solution. The evaluation was performed using the Beckman Coulter XLM flow cytometer (Winooski, VT, USA) and Flow Jo7 software (version 7.0 Copyright©; 2018 [or current year of copyright] Partek Inc., St. Louis, MO, USA).

### 4.5. In Silico Biopharmaceutical Study of the Compounds ***4a***–***c***, ***5a***–***c***

#### 4.5.1. Molecular Modeling of Compounds

The series of molecules were modeled in the Discovery Studio software (Dassault Systèmes BIOVIA, Discovery Studio Modeling Environment, Release 2017, San Diego, CA, USA: Dassault Systèmes, 2016), using carprofen as template compound. The analysis consisted of modeling 2D structures, adding specific substituents and completing valences with hydrogen atoms and passing these structures into three-dimensional space, in order to obtain 3D spatial structures.

#### 4.5.2. Energy Minimization

Minimization of these molecules was done using the Hamiltonian method: Forcefield MMFF94x at a gradient of 0.05. After calculating the minimum energy, the Gasteiger partial loads (PEOE) were applied.

#### 4.5.3. Determination of Drug-Like Character and Bioavailability

For establishing the pharmacological character of the newly synthesized compounds, the compliance with at least two Lipinski rules (drug-like nature) and with the Veber rule (bioavailability) was checked [29,30].

#### 4.5.4. Structural Similarities Using Chemical Compounds Database

By accessing the Expasy/medicinal chemistry database we identified the possible structural similarities of the analyzed compounds with other chemical compounds. 

#### 4.5.5. Identifying the Pharmacokinetic Profile

For the ADME prediction, a pkCSM platform was used [23], and for their toxicity, results from multiple database were compared [30,31].

We have selected the following properties: 1. Intestinal absorption, 2. blood–brain barrier permeability (BBB), 3. central nervous system permeability (CNS), 4. AMES toxicity, 5. maximum tolerated human dose, ability to inhibit human ether-a-gogo gene (hERG I and hERG II), 6. Lethal Dose 50 (on rat), 7. hepatotoxicity, 8. the ability of the substance to produce carcinogenic effects.

## Figures and Tables

**Table 1:** MIC (mg/mL) values for the compounds **4a**–**c**, **5a**–**d.**

	Compound	4a	4b	4c	5a	5b	5c	5d
Microorganism								
*Enterococcus faecalis* ATCC 29212		10	5	10	5	5	2.5	5
*Staphylococcus aureus* ATCC 25923		5	5	2.5	2.5	5	5	5
*Pseudomonas aeruginosa* ATCC 27853		2.5	2.5	2.5	2.5	2.5	2.5	2.5
*Escherichia coli* ATCC 25922		1.25	2.5	2.5	2.5	2.5	5	2.5
*Candida albicans* ATCC 90029		5	2.5	5	2.5	2.5	0.625	5

**Table 2:** Minimal bactericidal concentration (MBC) (mg/mL) values for the compounds **4a**–**c**, **5a**–**d**.

	Compound	4a	4b	4c	5a	5b	5c	5d
Microorganism								
*Enterococcus faecalis* ATCC 29212		10	10	10	10	10	5	5
*Staphylococcus aureus* ATCC 25923		5	5	5	5	5	5	5
*Pseudomonas aeruginosa* ATCC 27853		2.5	2.5	2.5	2.5	2.5	2.5	2.5
*Escherichia coli* ATCC 25922		1.25	5	5	5	5	5	5
*Candida albicans* ATCC 90029		10	5	10	5	5	0.625	10

**Table 3:** MBEC (mg/mL) values for the compounds **4a**–**c**, **5a**–**d**.

	Compound	4a	4b	4c	5a	5b	5c	5d
Microorganism								
*Enterococcus faecalis* ATCC 29212		2.5	0.009	2.5	1.25	0.078	10	0.625
*Staphylococcus aureus* ATCC 25923		1.25	0.625	1.25	0.312	0.039	1.25	0.312
*Pseudomonas aeruginosa* ATCC 27853		0.009	0.009	0.009	0.009	0.009	0.009	0.625
*Escherichia coli* ATCC 25922		1.25	1.25	1.25	10	1.25	2.5	1.25
*Candida albicans* ATCC 90029		0.312	0.039	0.625	0.078	1.25	0.312	0.156

**Table 4:** Lipinski and Veber rules validation, bioavailability score and predicted molecular weight (MW).

Compound	Lipinski Rule	Veber Rule	Bioavailability Score	MW (g/mol)
**4a**	YES	YES	0.55	391.85
**4b**	YES *	YES	0.55	459.85
**4c**	YES *	YES	0.55	426.30
**5a**	YES	YES	0.55	373.83
**5b**	YES *	YES	0.55	408.28
**5c**	YES	YES	0.55	441.83
**5d**	YES	YES	0.55	450.92
**Carprofen**	YES	YES	0.56	273.71

* **4b**, **4c**, **5b**, and **5d** don’t respect the LogP rule, being higher than 5, but they respect the other criteria of the Lipinski rule.

**Table 5:** Structural similarities.

Compound	Similar Compound	Percent	Chemical Structure	Target Structure of Similar Compound
**4a**	CHEMBL2171276 *	0.602	2-(6-chloro-9*H*-carbazol-2-yl)-*N*-phenylpropanamide *	Faah inhibition-fatty acid amide hydrolase (Norway rat)
	CHEMBL2171249	0.585		
	CHEMBL1316	0.585		
**4b**	CHEMBL2171276	0.557	2-(6-chloro-9*H*-carbazol-2-yl)-*N*-phenilpropanamide	Faah inhibition-fatty acid amide hydrolase (Norway rat)
**4c**	CHEMBL2171276 *	0.590	2-(6-chloro-9*H*-carbazol-2-yl)-*N*-phenilpropanamide	Faah inhibition-fatty acid amide hydrolase (Norway rat)
	CHEMBL2171249	0.573		
	CHEMBL1316	0.573		
**5a**	CHEMBL380021 *	0.595	2-[2-[5-[2-(4-chlorophenyl)ethyl]-1,3,4-oxadiazol-2-yl]-1~{*H*}-indol-4-yl]-~{*N*},~{*N*}-dimethylethanamine *	nociceptive receptor ligand
	CHEMBL2418797	0.588		
	CHEMBL197502	0.557		
**5b**	CHEMBL380021 *	0.594	2-[2-[5-[2-(4-clorofenil)etil]-1,3,4-oxadiazol-2-il]-1~{*H*}-indol-4-il]-~{*N*},~{*N*}-dimetiletanamine *	nociceptive receptor ligand
	CHEMBL2418797	0.576		
**5c**	CHEMBL2418770 *	0.587	2-(3~{*H*}-benzimidazol-5-yl)-5-[2-[4-(trifluoromethyl)phenyl]ethyl]-1,3,4-oxadiazole *	QPCT—glutaminyl-peptide cyclotransferase inhibition (human)
	CHEMBL380021	0.565		
**5d**	CHEMBL1611023	0.430	(4-[5-(4-Chlorobenzoyl)-1,3,4-oxadiazol-2-yl]pyridine)	inactive

* Ligand with higher percent of similarity with analyzed compound.

**Table 6:** Prediction of intestinal absorption, permeability of Caco2, blood–brain barrier (BBB) permeability, Renal OCT2.

Compound	4a	4b	4c	5a	5b	5c	5d	Carprofen	Unit
Intestinal absorption	91	89	90	94	92	91	96	94	% Absorption
Caco-2 pkCSM permeability	0.919	0.944	0.934	1.021	1.01	1.016	0.934	1.301	log Papp in 10^−6^ cm/s
BBB permeability	−0.635	−0.942	−0.816	0.428	0.383	0.342	−0.371	0.191	log BB
Renal Substrate OCT2	NO	NO	NO	NO	NO	NO	NO	NO	

**Table 7:** Toxicity descriptors.

Compound	4a	4b	4c	5a	5b	5c	5d	Unit
AMES toxicity	yes	yes	yes	yes	yes	yes	yes	Yes/No
DMT(human)	0.19	0.13	0.16	0.41	0.36	0.33	0.503	log mg/kg/day
I. hERGI	no	no	no	no	no	no	no	Yes/No
I. hERGII	yes	yes	yes	yes	yes	yes	yes	Yes/No
LD 50	2.46	2.46	2.50	2.30	2.33	2.34	2.60	mol/kg
Hepatotoxicity	yes	yes	yes	yes	yes	yes	yes	Yes/No

DMT—maximum tolerated human dose, I—inhibitor, LD—lethal dose.
